# Ablação da Fibrilação Atrial: Impacto da Ecocardiografia Intracardíaca na Redução do Tempo de Procedimento e Internação

**DOI:** 10.36660/abc.20220306

**Published:** 2023-05-02

**Authors:** Roberto Tofani Sant`Anna, Gustavo Glotz de Lima, Marco Aurélio Lumertz Saffi, Marcelo Lapa Kruse, Tiago Luiz Luz Leiria

**Affiliations:** 1 Instituto de Cardiologia Porto Alegre RS Brasil Instituto de Cardiologia , Porto Alegre , RS – Brasil; 2 Universidade Federal de Ciências da Saúde de Porto Alegre Porto Alegre RS Brasil Universidade Federal de Ciências da Saúde de Porto Alegre , Porto Alegre , RS – Brasil; 3 Hospital de Clínicas de Porto Alegre Porto Alegre RS Brasil Hospital de Clínicas de Porto Alegre , Porto Alegre , RS – Brasil

**Keywords:** Fibrilação Atrial/complicações, Ablação por Cateter, Ecocardiografia/métodos, Hospitalização, Veias Pulmonares/diagnóstico por imagem

## Abstract

**Fundamento:**

O ecocardiograma intracardíaco (EIC) permite visualizar estruturas cardíacas e reconhecer complicações durante a ablação da fibrilação atrial (AFA). Comparado ao ecocardiograma transesofágico (ETE), o EIC é menos sensível para detecção de trombo no apêndice atrial, porém requer mínima sedação e menos operadores, tornando-o atrativo num cenário de recursos restritos.

**Objetivo:**

Comparar 13 casos de AFA utilizando EIC (grupo AFA-EIC) com 36 casos de AFA utilizando ETE (grupo AFA-ETE).

**Método:**

Trata-se de corte prospectiva realizada em um único centro. O desfecho principal foi o tempo de procedimento. Desfechos secundários tempo de fluoroscopia, dose de radiação (mGy/cm2), complicações maiores e tempo de internação hospitalar em horas. O perfil clínico foi comparado pelo escore CHA2DS2-VASc. Um valor de p <0,05 foi considerado uma diferença estatisticamente significativa entre os grupos.

**Resultados:**

A mediana do escore de CHA2DS2-VASc score foi 1 (0-3) no grupo AFA-EIC e 1 (0-4) no grupo AFA-ETE. O tempo total de procedimento foi de 129 ± 27 min grupo AFA-EIC e 189 ± 41 no AFA-ETE (p<0,001); o grupo AFA-EIC recebeu uma dose menor de radiação (mGy/cm2, 51296 ± 24790 vs. 75874 ± 24293; p=0,002), no entanto, o tempo de fluoroscopia em minutos mostrou-se semelhante (27,48 ± 9,79 vs. 26,4 ± 9,32; p=0,671). As medianas do tempo de hospitalização não se mostraram diferentes, 48 (36-72) horas (AFA-EIC) e 48 (48-66) horas (AFA-ETE) (p=0,27).

**Conclusão:**

Nesta coorte, AFA-EIC foi relacionado a menores tempos de procedimento e menor exposição à radiação, sem aumentar o risco de complicações ou o tempo de internação hospitalar.

## Introdução

A fibrilação atrial (FA) é a arritmia cardíaca mais comum, afetando mais de 37 milhões de pessoas em todo o mundo. ^1^

A ablação da FA, obtida pelo isolamento das veias pulmonares (IVP), é recomendada pelas diretrizes das sociedades profissionais como uma terapia útil para manter o ritmo sinusal e melhorar a qualidade de vida. ^2 , 3^

O sucesso e a segurança da IVP estão na dependência da identificação adequada dos pontos anatômicos por métodos de imagem, que incluem mapeamento eletroanatômico, tomografia computadorizada, ressonância magnética cardíaca e ecocardiograma. ^4^

A utilização do ecocardiograma durante a ablação de FA (AFA) tem várias vantagens como alta resolução em tempo real das estruturas cardíacas e a disponibilidade. O ecocardiograma também tem o potencial de aumentar a segurança do paciente pela identificação precoce de complicações relacionadas ao procedimento e de reduzir a exposição à radiação do paciente e da equipe ao diminuir a dependência da fluoroscopia. ^5^

Duas formas de ecocardiografia têm sido utilizadas na cardiologia intervencionista: transesofágica (ETE) e intracardíaca (EIC). O ETE oferece imagens superiores em resolução e menor custo inicial, pois a sonda é reutilizável. No entanto, requer anestesia geral e profissionais treinados, além de acarretar risco de trauma ao trato gastrointestinal. Tomados em conjunto, esses fatores podem aumentar o custo total do procedimento, principalmente em cenários de recursos humanos restritos. Existem poucas comparações diretas dos dois métodos para IVP. O objetivo deste estudo foi comparar o IVP usando ETE com o IVP usando EIC quanto ao tempo de procedimento, complicações maiores, exposição à radiação e tempo de internação hospitalar.

## Métodos

### Desenho e aprovação ética

Este foi um estudo de coorte prospectivo de um único centro de pacientes recrutados consecutivamente no Serviço de Eletrofisiologia entre dezembro de 2017 e junho de 2021. O protocolo foi elaborado de acordo com a declaração *STROBE* para relatar estudos observacionais ^6^ e foi aprovado pelo Comitê de Ética local (registro UP 5252/16).

### Amostra

Todos os pacientes incluídos tinham indicação classe I ou IIA para AFA de acordo com as diretrizes internacionais. ^2 , 3^

Foram incluídos pacientes com FA paroxística e FA persistente com menos de 1 ano de evolução. Excluímos pacientes com trombo intracavitário no ETE realizado no dia da AFA.

### Procedimento e acompanhamento

Todos os procedimentos foram realizados pelo mesmo eletrofisiologista, com o paciente sob anestesia geral com utilização de um termômetro esofágico ao nível do átrio esquerdo (AE). Após as punções venosas, um cateter decapolar foi posicionado no seio coronário. Duas punções transseptais foram realizadas guiadas pelo ecocardiograma com bainha SL-1 e agulha BRK-1 *(St. Jude Medical, St Paul, EUA)* seguidas de heparinização terapêutica. Um cateter duodecapolar circular *(Biosense Webster, Irvine, EUA)* foi usado para mapear o AE e um cateter de ablação com ponta irrigada foi introduzido no AE. O mapeamento atrial foi realizado pelo sistema tridimensional *EnSite (St. Jude Medical, St. Paul, EUA)* . A radiofrequência foi aplicada circunferencialmente ao redor das veias pulmonares ipsilaterais até que o isolamento elétrico fosse alcançado. No pós-operatório imediato, os esquemas de terapia anticoagulante foram iniciados 6 horas após a retirada das bainhas introdutoras e mantidos por, no mínimo, 60 dias. Todos os pacientes foram mantidos com pantoprazol, inibidor da bomba de prótons, por 30 dias, e antiarrítmico (usado previamente no pré-operatório) por 90 dias.

Os pacientes foram divididos em dois grupos de acordo com o método de imagem utilizado durante a AFA: grupo ecocardiograma intracardíaco (AFA-EIC) ou grupo ecocardiograma transesofágico (AFA-ETE). No grupo AFA-EIC, um cateter de imagem por ultrassom *phased-array* de 10 Fr (AcuNav, Acuson) foi introduzido através de uma bainha de 11 Fr através da veia femoral esquerda. O EIC foi usado para garantir o posicionamento do cateter, local apropriado de fornecimento de energia, monitoramento da formação de microbolhas e detecção de complicações. No grupo AFA-ETE, o ecocardiograma transesofágico foi realizado por especialista em imagem cardíaca. A escolha entre os métodos de imagem teve lugar com base na disponibilização ou não de ecocardiograma intracardíaco pela fonte seguradora de saúde do paciente em questão. Com relação ao aparelho de RX, foi utilizado um equipamento *PHILIPS ALLURA XPER* , sendo configurado na taxa de 3.75 quadros por segundo.

Os pacientes foram admitidos para monitoramento na unidade de terapia intensiva (UTI) por um período mínimo de uma noite após o procedimento de ablação. Depois disso, a alta foi de acordo com o médico assistente. As drogas antiarrítmicas foram mantidas por pelo menos 3 meses. Todos os pacientes incluídos neste estudo foram acompanhados por pelo menos três meses (tempo médio: 650,95 ± 380,86 dias).

### Desfechos

O desfecho primário foi o tempo total do procedimento. Os desfechos secundários foram: complicações maiores (tamponamento cardíaco, acidente vascular cerebral, perfuração esofágica), tempo de fluoroscopia, dose de radiação em mGy/cm ^2^ e tempo de alta hospitalar medido em horas. O perfil clínico foi comparado pelo escore *CHA2DS2-VASc* e por parâmetros ecocardiográficos de fração de ejeção do ventrículo esquerdo (FEVE) e diâmetro do átrio esquerdo (DAE).

### Análise estatística

As análises estatísticas foram realizadas com o software *Statistical Package for the Social Sciences* (SPSS) versão 22.0 *(SPSS Inc., Chicago, IL, EUA)* . As variáveis contínuas com distribuição normal estão descritas através de média e desvio padrão e as variáveis contínuas sem distribuição normal estão descritas através de mediana e intervalo interquartil. O teste estatístico para avaliar a normalidade foi o *Shapiro-Wilk* . O teste T de *Student* de amostras independentes foi usado para as variáveis com distribuição normal e o teste não paramétrico *U de Mann-Whitney* para variáveis sem distribuição normal. As variáveis categóricas estão expressas como frequências e porcentagens e foram comparadas através do teste χ2 ou exato de Fisher. Um valor de p <0,05 foi considerado uma diferença estatisticamente significativa entre os grupos.

## Resultados

### Perfil clínico

Um total de 49 pacientes foi submetido a IVP, sendo a maioria do sexo masculino (81%) com média de idade de 54 ± 12,2 anos. A comorbidade mais comum foi a hipertensão, identificada em 65,3% dos pacientes incluídos. Em geral, os pacientes apresentavam poucas comorbidades, refletidas por um escore *CHADS2Vasc* mediano de 1 em ambos os grupos. A maioria dos pacientes apresentou FA paroxística (89,8%), com prevalência semelhante entre os grupos AFA-ETE (84,6%) e AFA-EIC (91,6%). Betabloqueadores (59,1%) e amiodarona (57,1%) foram os antiarrítmicos mais prescritos. Cerca de um quarto dos pacientes incluídos estavam recebendo uma combinação de medicamentos (28,5%).

A Tabela 1 apresenta as características demográficas e clínicas basais, escores clínicos e parâmetros ecocardiográficos para as populações agrupadas conforme o procedimento foi auxiliado por EIC ou ETE.

**Tabela 1:** – Características clínicas e demográficas dos pacientes incluídos. Porto Alegre, RS

Variável	AFA-EIC (n=13)	AFA-ETE (n=36)	Valor p
Idade*	52,4 ± 11	54,7 ± 12,3	0,57
Sexo masculino†	10(77)	30(83,3)	0,6
Peso*	83,6 ± 17	90,4 ± 11,8	0,24
Insuficiência cardíaca†	1(7,7)	5(13,8)	0,53
Hipertensão†	9(69,2)	24(66,6)	0,81
Diabetes†	1(7,7)	6(16,6)	0,41
AVC/ AIT†	-	-	-
FA persistente†	2(15,3)	3(8,6)	0,6
Doença vascular†	2(15,4)	3(8,6)	0,49
Escore CHA _2_ DS _2_ -VASc‡	1(0-3)	1(0-4)	0,52
0	3(23,1)	9(25)	
1	4(30,8)	11(30,5)	
2	5(38,5)	6(16,6)	
3	1(7,7)	4(11,1)	
4	-	5(13,8)	
FEVE (%)*	67 ± 2,7	68 ± 4	0,41
Diâmetro AE (mm)*	42 ± 0,5	42 ± 2	0,80

* Dados apresentados em média *+* desvio padrão; † Frequência absoluta e relativa; ‡medianas e intervalo interquatílico (percentis 25 e 75). AE: átrio esquerdo; AFA: ablação de fibrilação atrial; AIT: ataque isquêmico transitório; AVC: acidente vascular isquêmico; EIC: ecocardiograma intracardíaco; ETE: ecocardiograma transesofágico; FEVE: fração de ejeção do ventrículo esquerdo.

A Tabela 2 mostra os fármacos anticoagulantes e antiarrítmicos de acordo com o grupo. A figura central destaca os principais resultados do estudo.

**Tabela 2:** – Utilização de anticoagulantes e antiarrítmicos conforme grupo. Porto Alegre, RS

Variável	AFA-EIC (n=13)	AFA-ETE (n=36)
Anticoagulação*		
Nenhum	8(61,5)	16(44,4)
Varfarina	-	1(2,8)
NOAC	5(38,4)	18(50)
Heparina	-	1(2,8)
Antiarrítmicos*		
Betabloqueador	8(61,5)	21(58,3)
Amiodarona	7(53,8)	21(58,3)
Propafenona	3(23)	2(5,5)
Digoxina	1(7,7)	-
Combinação	5(38,4)	8(22,2)

* Dados apresentados em frequência absoluta e relativa. AFA: ablação de fibrilação atrial; EIC: ecocardiograma intracardíaco; ETE: ecocardiograma transesofágico.

**Figura Central:**
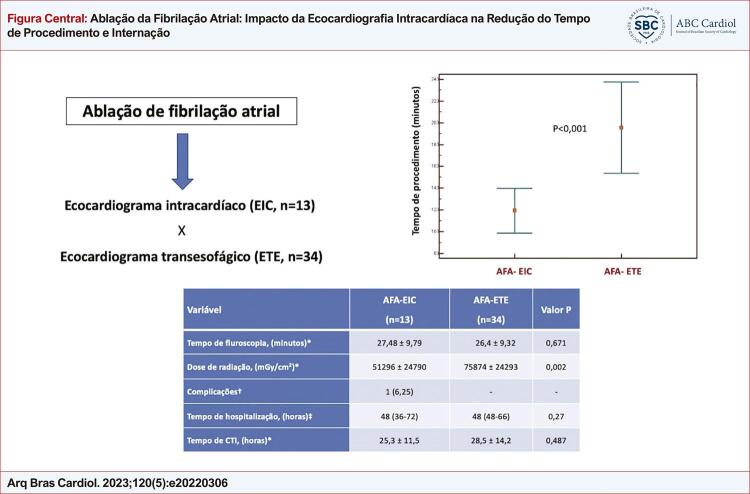
: Ablação da Fibrilação Atrial: Impacto da Ecocardiografia Intracardíaca na Redução do Tempo de Procedimento e Internação

### Procedimento de ablação e acompanhamento

Dois pacientes do grupo AFA-ETE foram excluídos da análise dos resultados do procedimento e de acompanhamento devido à indisponibilidade do operador do ecocardiograma transesofágico durante a totalidade da ablação, de forma a manter as comparações entre os tempos de procedimento fidedignas. O tempo total do procedimento foi de 129 ± 27 minutos no grupo AFA-EIC e 189 ± 41 minutos no AFA-ETE (p<0,001). O grupo AFA-EIC também recebeu menor dose de radiação (mGy/cm ^2^ , 51296 ± 24790 *vs.* 75874 ± 24293; p=0,002), apesar de apresentar tempo de fluoroscopia semelhante (minutos, 27,48 ± 9,79 *vs.* 26,4 ± 9,32; p=0,671).

A taxa de complicações maiores foi baixa e semelhante entre os dois grupos. Não houve casos de tamponamento cardíaco, eventos cerebrais ou esofágicos. Um paciente do grupo AFA-EIC teve oclusão da artéria femoral superficial direita diagnosticada no segundo dia pós-procedimento e tratado com sucesso com embolectomia. Um paciente do AFA-ETE apresentou evolução acelerada da tireoidite de *Hashimoto* , provavelmente relacionada à amiodarona. Três pacientes foram submetidos a um segundo procedimento de IVP durante o seguimento. Os demais dados são mostrados na tabela 3 .

**Tabela 3:** – Resultado do isolamento das veias pulmonares. Porto Alegre, RS

Variável	AFA-EIC (n=13)	AFA-ETE (n=34)	Valor p
Tempo de procedimento, (minutos)*	129 ± 27	189 ± 41	<0,001
Tempo de fluoroscopia, (minutos)*	27,48 ± 9,79	26,4 ± 9,32	0,671
Dose de radiação, (mGy/cm ^2^ )*	51296 ± 24790	75874 ± 24293	0,002
Complicações†	1 (6,25)	-	-
AVC	-	-	-
Tamponamento	-	-	-
Sangramento maior	-	-	-
Perfuração esofágica	-	-	-
Complicações vasculares†	1 (6,25)	-	-
Tempo de hospitalização, (horas)‡	48 (36-72)	48 (48-66)	0,27
Tempo de CTI, (horas)*	25,3 ± 11,5	28,5 ± 14,2	0,487

* Dados apresentados em média *+* desvio padrão; † Frequência absoluta e relativa; ‡medianas e intervalo interquatílico (percentis 25 e 75). AFA: ablação de fibrilação atrial; AVC: acidente vascular isquêmico; CTI: centro de tratamento intensivo; EIC: ecocardiograma intracardíaco; ETE: ecocardiograma transesofágico; mGy/cm ^2^: Centímetro quadrado miligray.

## Discussão

Nosso estudo mostra que o IVP auxiliado por EIC tem menor tempo de procedimento e exposição à radiação quando comparado à assistência feita através de ETE, mantendo a segurança do procedimento. O tempo de internação e o tempo de permanência na UTI foram semelhantes entre os dois grupos. Os grupos apresentavam perfil clínico similar, conforme demonstrado pela mediana do escore *CHADS2Vasc* de 1 em ambos os grupos e FEVE e DAE semelhantes.

A identificação de pontos anatômicos é de suma importância para uma ablação de FA bem-sucedida. Em um estudo de Toffanin et al., ^7^ avaliando a anatomia da VP com ETE e angiorressonância magnética, apenas 42% dos pacientes apresentavam anatomia normal das veias pulmonares com duas veias direita e esquerda. A integração de imagens com tomografia computadorizada ou ressonância magnética cardíaca pode melhorar a taxa de sobrevida livre de FA após ablação. ^8 , 9^ Curiosamente, os tempos fluoroscópicos geralmente não são menores em tais estudos.

Encontramos uma redução acentuada no tempo total do procedimento. Atribuímos essa diferença principalmente ao tempo maior para posicionar a sonda transesfoágica e a obtenção de imagens mais adequadas de forma imediata pelo operador ao utilizar o EIC. O ETE dependia de um profissional extra que precisava colocar adequada proteção contra a radiação e reposicionar a sonda com imagens que satisfizessem o operador não apenas no momento da punção transeptal, mas também na suspeita de complicações e ao final do procedimento para descartar a presença de tamponamento. Em dois casos da série, o profissional responsável pelo ETE precisou ser aguardado na sala, pois foi requisitado de urgência em outro procedimento. Optamos por excluir esses pacientes das comparações. Em dois casos em especial, o ETE não conseguiu identificar adequadamente agulha de punção nem a fossa *ovalis* , aumentando o tempo de procedimento. Outros fatores incluem a segurança e independência do exame ser realizado pelo mesmo operador que está realizando a ablação e a necessidade da intubação esofágica no caso do ETE. O tempo total de fluoroscopia foi semelhante entre os grupos, pois os fatores que prolongaram o tempo de procedimento com uso do ETE não podem ser compensados com maior utilização de raio X. Na metanálise de Isath et al., ^10^ que reuniu 19 estudos e comparou os resultados de ablações realizadas com ou sem EIC, houve uma redução tanto do tempo de fluoroscopia quanto do tempo de procedimento, mas deve ser considerado que não foi feita uma comparação direta com ETE e sim com fluoroscopia isolada e/ou mapeamento eletroanatômico.

O ETE é o padrão-ouro para identificação do trombo do apêndice do AE. ^4^ A ETE é um método invasivo com risco de complicação de aproximadamente 0,9%. ^11 , 12^ O EIC pode melhorar o resultado do procedimento reduzindo o risco de recorrência de FA e o risco de estenose da veia pulmonar, ^13^ apesar de sua limitação na identificação adequada de pequenos ramos das veias pulmonares. Isath et al., ^10^ em uma análise de 14 anos reunindo diversos centros estado-unidenses, demonstraram que o uso de EIC durante a AFA estava relacionado a menor mortalidade intra-hospitalar, menor risco de complicações do procedimento e menor tempo de internação. Especificamente, o EIC permite a detecção mais rápida de complicações, incluindo derrame pericárdico, embolia aérea, disfunção ventricular como causa de hipotensão e a formação de trombos nas bainhas e nos cateteres utilizados para ablação.

Uma metanálise de Goya et al., ^14^ avaliou os resultados com o uso de EIC para ablação de arritmia. Dos 19 estudos incluídos, 14 foram realizados em pacientes com FA. A utilização de EIC foi associada a um tempo de fluoroscopia significativamente menor, dose de fluoroscopia e menor tempo de procedimento, sem comprometer a eficácia clínica ou a segurança do procedimento. Embora os achados sejam semelhantes ao nosso estudo, deve-se notar que a metanálise de Goya et al., ^14^ não foi uma comparação direta entre EIC e ETE. O grupo comparador foi amplo e incluiu mapeamento eletroanatômico, fluoroscopia ou outras modalidades de imagem, por vezes utilizadas de forma conjunta ou nada além da fluoroscopia. Ribeiro et al., ^15^ comparando a oclusão percutânea do átrio esquerdo guiada por EIC com ETE, encontraram tempo de procedimento, risco de complicações e tempo de procedimento semelhantes entre os dois métodos.

Existe uma relação linear entre a dose de radiação e o risco de malignidade futura. A exposição à radiação também está ligada a lesões cutâneas agudas, distúrbios da tireoide, catarata, entre outras doenças. ^16 , 17^ Uma vez que nenhuma dose de radiação é considerada segura e os operadores podem estar em maior risco devido aos múltiplos procedimentos que realizam, recomenda-se que a exposição à radiação seja tão baixa quanto possível. ^17^ Nesse sentido, a dose significativamente menor à qual os pacientes do grupo EIC foram submetidos é um ponto a favor do método. Acreditamos que o EIC tenha permitido utilizar imagens com menor intensidade, o que permitiu redução da dose de radiação, apesar dos tempos similares de fluoroscopia.

Ao exigir um acesso vascular adicional de bainha de 11 Fr, o EIC pode aumentar o risco de complicações vasculares. Este não foi o caso em nosso estudo. Na metanálise realizada por Goya et al., ^14^ houve tendência de aumento de complicações vasculares com EIC (RR 1,93; IC 95%, 0,81-4,60; p = 0,14).

O EIC requer treinamento apropriado do operador em manipulação de cateter e interpretação de imagem. Por ser progressivamente adotado em outras formas de ablação, ^18 , 19^ acreditamos que deve fazer parte das habilidades dos eletrofisiologistas que lidem com ablação complexas. O EIC também requer um cateter não reutilizável, aumentando o custo do procedimento, que é pelo menos parcialmente compensado pela redução do número de funcionários necessários para o procedimento, bem como pela redução do consumo de medicação anestésica. ^20^

O EIC permite a visualização em tempo real das estruturas cardíacas, avaliação intraprocedimento da interface cateter-tecido e complicações rápidas. Nossa amostra foi insuficiente para comparar a eficácia do procedimento entre os dois métodos. Em um estudo do mundo real, a ablação de taquicardia ventricular usando EIC foi associada a uma menor probabilidade de reinternação relacionada por taquicardia ventricular em 12 meses e de ablação repetida em comparação com pacientes em que o EIC não foi empregado. ^18^ Um estudo retrospectivo de pacientes com taxa por serviço do *Medicare* submetidos a ablação por cateter para fibrilação atrial observou que o uso de EIC estava associado a menor risco de ablação repetida (taxa de risco 0,59; IC 95% 0,37-0,92). ^21^

### Limitações do estudo

Nosso estudo sofre de algumas limitações. (1) Tamanho amostral pequeno, o que pode ser responsável por não ter detectado algumas diferenças entre os grupos, como o tempo de internação. (2) Falta de randomização. A utilização do EIC foi baseada na disponibilidade de seguro saúde, o que poderia influenciar sistematicamente no perfil dos pacientes de cada grupo. A Tabela 1 mostra que esse grupo apresentou perfil muito semelhante quantos aos principais fatores de risco para recorrência de FA e para maior dificuldade técnica durante a ablação, incluindo tamanho de átrio esquerdo, o que mitiga essa limitação. Algumas das explicações para diferença entre o tempo de procedimento entre os grupos, como o tempo para o operador do ETE se disponibilizar, são devidas a fatores locais e podem não refletir a realidade de outras instituições. (3) Não realizamos comparação direta de custos entre as duas estratégias. Como os pacientes possuíam planos de saúde diferentes, a forma de reembolso e o custo variavam, impedindo uma análise homogênea do efeito do método utilizado no custo. O ecocardiograma intracardíaco é vendido para nossa instituição por R$ 7.500,00. Em relação ao ETE, o tempo de procedimento em média 55 minutos maior levaria a uma elevação de custo com sala de R$ 1.100,00, enquanto o profissional para realização do exame teria um custo médio R$ 1.000,00. Não é possível estimar o custo indireto dessa maior ocupação de recursos de saúde, especialmente durante a pandemia, período em que foram realizadas 25 das 49 ablações analisadas no estudo.

## Conclusão

Em nossa coorte, o uso de AFA com EIC foi relacionado a menores tempos de procedimento e menor exposição à radiação, sem aumentar o risco de complicações ou tempo de internação hospitalar. O aumento do custo inicial do procedimento pode ser compensado por uma menor ocupação da sala de operação e pela necessidade de menos funcionários treinados para o procedimento.
