# Supplementary material for: Ablação da Fibrilação Atrial: Impacto da Ecocardiografia Intracardíaca na Redução do Tempo de Procedimento e Internação
Source: Arq Bras Cardiol. 2023 May 2;120(5):e20220306. [Article in Portuguese] doi: 10.36660/abc.20220306 (PMC10263395; doi:10.36660/abc.20220306)
Supplement: Translation

# Translation: Atrial Fibrillation Ablation: Impact of Intracardiac Echocardiography in Reducing Procedure Time and Hospitalization

**Authors:** Roberto Tofani Sant`Anna, Gustavo Glotz de Lima, Marco Aurélio Lumertz Saffi, Marcelo Lapa Kruse, Tiago Luiz Luz Leiria

**Affiliations:** 1 Brazil Instituto de Cardiologia, Porto Alegre, RS – Brazil; 2 Brazil Universidade Federal de Ciências da Saúde de Porto Alegre, Porto Alegre, RS – Brazil; 3 Brazil Hospital de Clínicas de Porto Alegre, Porto Alegre, RS – Brazil

**Keywords:** Atrial Fibrillation/complications, Catheter Ablation, Echocadiography/methods, Hospitalization, Pulmonary Veins/diagnostic imaging

## Abstract

**Background:**

Intracardiac echocardiography (ICE) allows visualization of cardiac structures and recognition of complications during atrial fibrillation ablation (AFA). Compared to transesophageal echocardiography (TEE), ICE is less sensitive to detecting thrombus in the atrial appendage but requires minimal sedation and fewer operators, making it attractive in a resource-constrained setting.

**Objective:**

To compare 13 cases of AFA using ICE (AFA-ICE group) with 36 cases of AFA using TEE (AFA-TEE group).

**Methods:**

This is a single-center prospective cohort study. The main outcome was procedure time. Secondary outcomes: fluoroscopy time, radiation dose (mGy/cm2), major complications, and length of hospital stay in hours. The clinical profile was compared using the CHA2DS2-VASc score. A p-value <0.05 was considered a statistically significant difference between groups.

**Results:**

The median CHA2DS2-VASc score was 1 (0-3) in the AFA-ICE group and 1 (0-4) in the AFA-TEE group. The total procedure time was 129 ± 27 min in the AFA-ICE group and 189 ± 41 min in the AFA-TEE group (p<0.001); the AFA-ICE group received a lower dose of radiation (mGy/cm2, 51296 ± 24790 vs. 75874 ± 24293; p=0.002), despite the similar fluoroscopy time (27.48 ± 9. 79 vs. 26.4 ± 9.32; p=0.671). The median length of hospital stay did not differ; 48 (36-72) hours (AFA-ICE) and 48 (48-66) hours (AFA-TEE) (p=0.27).

**Conclusions:**

In this cohort, AFA-ICE was related to shorter procedure times and less exposure to radiation without increasing the risk of complications or the length of hospital stay.

## Introduction

Atrial fibrillation (AF) is the most common cardiac arrhythmia, affecting more than 37 million people worldwide.^1^AF ablation, achieved by pulmonary vein isolation (PVI), is recommended by the guidelines of professional societies as a useful therapy to maintain sinus rhythm and improve quality of life.^2 , 3^The success and safety of PVI depend on the proper identification of anatomical landmarks by imaging methods, which include electro-anatomical mapping, computed tomography, cardiac magnetic resonance, and echocardiogram.^4^

The use of echocardiography during AF ablation (AFA) has several benefits, such as the high real-time resolution of cardiac structures and availability. Echocardiography also has the potential to increase patient safety by early identification of procedure-related complications and to reduce patient and staff radiation exposure by decreasing dependency on fluoroscopy.^5^

Two forms of echocardiography have been used in interventional cardiology: transesophageal (TEE) and intracardiac (ICE). TEE offers a better image resolution and lower initial cost because the probe is reusable. However, it requires general anesthesia and additionally trained professionals and carries a risk of trauma to the gastrointestinal tract. Together, these factors can increase the total cost of the procedure, particularly in a constrained human resource setting. There are few direct comparisons of the two methods for PVI. This study aimed to compare PVI using TEE with PVI using ICE regarding procedure time, major complications, radiation exposure, and length of hospital stay.

## Methods

### Study design

This was a single-center prospective cohort study of patients consecutively recruited from the Electrophysiology Service between December 2017 and June 2021. The protocol was designed following the STROBE statement for reporting observational studies^6^and was approved by the local Ethics Committee (registration UP 5252/16).

### Sample

All patients included in the study had a class I or IIA indication for AFA according to international guidelines.^2 , 3^We included patients with paroxysmal and persistent AF with less than 1 year of evolution. We excluded patients who presented with intracavitary thrombus on TEE performed on the day of the AFA.

### Procedure and follow-up

All procedures were performed by the same electrophysiologist, with the patient under general anesthesia using an esophageal thermometer at the level of the left atrium (LA). After venipunctures, a decapolar catheter was placed in the coronary sinus. Two transseptal punctures were performed, guided by echocardiography with an SL-1 sheath and a BRK-1 needle *(St. Jude Medical, St Paul, USA),* followed by therapeutic heparinization. A circular duodecapolar catheter *(Biosense Webster, Irvine, USA)* was used to map the LA, and an irrigated tip ablation catheter was placed in the LA. Atrial mapping was performed using the three-dimensional EnSite system *(St. Jude Medical, St. Paul, USA)* . Radiofrequency was applied circumferentially around the ipsilateral pulmonary veins until electrical isolation was achieved. Anticoagulant therapy was initiated 6 hours after removal of the sheaths and maintained for at least 60 days. All patients received pantoprazole, a proton pump inhibitor, for 30 days and were maintained with the same antiarrhythmic drug used preoperatively for 90 days.

Patients were divided into two groups according to the imaging method used during AFA: intracardiac echocardiogram (AFA-ICE) group or transesophageal echocardiogram (AFA-TEE) group. In the AFA-ICE group, a 10 Fr phased-array ultrasound imaging catheter (AcuNav, Acuson) was introduced through an 11 Fr sheath through the left femoral vein. The ICE was used to ensure catheter placement, proper energy delivery site, monitoring of microbubble formation, and detection of complications. In the AFA-TEE group, the transesophageal echocardiogram was performed by a specialist in cardiac imaging. The choice between the imaging methods was based on whether an intracardiac echocardiogram was available from the health insurance source of the patient in question. Regarding the X-ray device for fluoroscopy, *PHILIPS ALLURA XPER* equipment was configured at a rate of 3.75 frames per second.

Patients were admitted for monitoring in the intensive care unit (ICU) for at least one night after the ablation procedure. After that, discharge was at the discretion of the attending physician. Antiarrhythmic drugs were maintained for at least 3 months. All patients included in this study were followed up for at least three months (mean time: 650.95 ± 380.86 days).

### Outcomes

The primary outcome was the total procedure time. Secondary outcomes were major complications (cardiac tamponade, stroke, esophageal perforation), fluoroscopy time, radiation dose in mGy/cm^2^, and time to hospital discharge measured in hours. The clinical profile was compared using the CHA2DS2-VASc score and echocardiographic parameters of left ventricular ejection fraction (LVEF) and left atrial diameter (LAD).

### Statistical analysis

Statistical analyzes were performed using the Statistical Package for the Social Sciences (SPSS) version 22.0 software *(SPSS Inc., Chicago, IL, USA)* . Continuous variables with normal distribution are described as mean, and standard deviation, and continuous variables without normal distribution are described as median and interquartile range. The statistical test to assess normality was the Shapiro-Wilk. We used Student’s t-test for independent samples with normal distribution and the non-parametric Mann-Whitney U test for variables without normal distribution. Categorical variables are expressed as frequencies and percentages and were compared using the χ2 test or Fisher’s exact test. A p-value <0.05 was considered a statistically significant difference between groups.

## Results

### Clinical profile

Forty-nine patients underwent PVI, the majority male (81%) with a mean age of 54 ± 12.2 years. The most common comorbidity was hypertension, identified in 65.3% of the included patients. Patients had few comorbidities, as reflected by a median CHADS2Vasc score of 1 in both groups. Most patients had paroxysmal AF (89.8%), with a similar prevalence between the AFA-TEE (84.6%) and AFA-ICE (91.6%) groups. Beta-blockers (59.1%) and amiodarone (57.1%) were the most prescribed antiarrhythmics. About a quarter of the patients included were receiving a combination of medications (28.5%).

Table 1 presents the baseline demographic and clinical characteristics, clinical scores, and echocardiographic parameters for the populations grouped according to whether ICE or TEE aided the procedure.

**Table 1:** – Clinical and demographic characteristics of included patients. Porto Alegre, RS

Variable	AFA-ICE (n=13)	AFA-TEE (n=36)	p value
Age*	52.4 ± 11	54.7 ± 12.3	0.57
Male sex†	10(77)	30(83.3)	0.6
Weigth*	83.6 ± 17	90.4 ± 11.8	0.24
Heart failure†	1(7.7)	5(13.8)	0.53
Hypertension†	9(69.2)	24(66.6)	0.81
Diabetes†	1(7.7)	6(16.6)	0.41
Stroke/transient ischemic attack†	-	-	-
Persistent AF†	2(15.3)	3(8.6)	0.6
Vascular disease†	2(15.4)	3(8.6)	0.49
CHA_2_DS_2_-VASc score‡	1(0-3)	1(0-4)	0.52
0	3(23.1)	9(25)	
1	4(30.8)	11(30.5)	
2	5(38.5)	6(16.6)	
3	1(7.7)	4(11.1)	
4	-	5(13.8)	
LVEF (%)*	67 ± 2.7	68 ± 4	0.41
LA diameter (mm)*	42 ± 0.5	42 ± 2	0.80

* Data presented as mean + standard deviation; † Absolute and relative frequency; ‡ Medians and interquartile range (25th and 75th percentiles); ICE: Intracardiac echocardiography; TEE: transesophageal echocardiography; AF: atrial fibrillation; LVEF: left ventricular ejection fraction; LA: left atrium.

Table 2 shows anticoagulant and antiarrhythmic drugs according to the group. The central illustration highlights the main results of the study.

**Table 2:** – Use of anticoagulants and antiarrhythmics according to the group. Porto Alegre, RS

Variable	AFA-ICE (n=13)	AFA-TEE (n=36)
Anticoagulation*		
None	8(61.5)	16(44.4)
Warfarin	-	1(2.8)
NOAC	5(38.4)	18(50)
Heparin	-	1(2.8)
Antiarrhythmics*		
Beta-blocker	8(61.5)	21(58.3)
Amiodarone	7(53.8)	21(58.3)
Propafenone	3(23)	2(5.5)
Digoxin	1(7.7)	-
Combination	5(38.4)	8(22.2)

* Data presented as absolute and relative frequency. ICE: Intracardiac echocardiography; TEE: transesophageal echocardiography; NOAC: Novel Oral Anticoagulants for Atrial Fibrillation.

**Central Illustration:** : Atrial Fibrillation Ablation: Impact of Intracardiac Echocardiography in Reducing Procedure Time and Hospitalization

### Ablation procedure and follow-up

In order to maintain a reliable comparison of the procedure times between the two groups, we excluded from the analysis two cases of the AFA-TEE group in which the transesophageal echocardiogram operator was not present on site for the entirety of the procedure. The total procedure time was 129 ± 27 minutes in the AFA-ICE group and 189 ± 41 minutes in the AFA-ETE group (p<0.001). The AFA-ICE group also received a lower dose of radiation (mGy/cm^2^, 51296 ± 24790 vs. 75874 ± 24293; p=0.002), despite having a similar fluoroscopy time (minutes, 27.48 ± 9.79 vs. 26.4 ± 9.32; p=0.671).

The rate of major complications was low and similar between the two groups. There were no cases of cardiac tamponade or cerebral or esophageal events. One patient in the AFA-ICE group had an occlusion of the right superficial femoral artery diagnosed on the second day after the procedure and successfully treated with embolectomy. One patient from the AFA-ETE had an accelerated evolution of Hashimoto’s thyroiditis, probably related to amiodarone. Three patients underwent a second PVI procedure during follow-up. Additional relevant data are shown in table 3 .

**Table 3:** – Pulmonary vein isolation outcome. Porto Alegre, RS

Variable	AFA-ICE (n=13)	AFA-TEE (n=34)	p value
Procedure time (minutes)*	129 ± 27	189 ± 41	<0.001
Fluoroscopy time (minutes)*	27.48 ± 9.79	26.4 ± 9.32	0.671
Radiation dose (mGy/cm^2^)*	51296 ± 24790	75874 ± 24293	0.002
Complications†	1 (6.25)	-	-
Stroke	-	-	-
Tamponade	-	-	-
Major bleeding	-	-	-
Esophageal perforation	-	-	-
Vascular complications†	1 (6.25)	-	-
Hospitalization time (hours)‡	48 (36-72)	48 (48-66)	0.27
ICU time (hours)*	25.3 ± 11.5	28.5 ± 14.2	0.487

* Data presented as mean *+* standard deviation; † Absolute and relative frequency; ‡ Medians and interquartile range (25th and 75th percentiles); ICE: Intracardiac echocardiography; TEE: transesophageal echocardiography; ICU: intensive care unit.

## Discussion

Our study shows that ICE-assisted PVI is associated with a shorter procedure time and lower radiation exposure when compared to TEE-assisted PVI while maintaining the procedure’s safety. The duration of both hospitalization and ICU stay were similar between groups. The two groups also had an equivalent clinical profile, as demonstrated by a median CHADS2Vasc score of 1 in both groups and similar LVEF and AED.

Identification of anatomical landmarks is of paramount importance for successful AF ablation. In a study by Toffanin et al.,^7^evaluating the anatomy of the PV with TEE and magnetic resonance, only 42% of the patients had normal anatomy of the pulmonary veins with two right and left veins. Integration of images with computed tomography or cardiac magnetic resonance imaging may improve AF-free survival rate after ablation.^8 , 9^Interestingly, fluoroscopic times are generally not shorter in such studies.

We found a marked reduction in total procedure time. We attribute this difference mainly to the longer time to position the transesophageal probe and the operator’s ability to obtain adequate images when using the EIC immediately. TEE depended on an extra professional who needed to put on adequate protection against radiation and to reposition the probe to provide adequate images for the operator, not only at the time of transseptal puncture but also when complications were suspected and at the end of the procedure to rule out the presence of tamponade. In two cases of our series, the professional responsible for the TEE had to have waited in the room as he was urgently requested for another procedure. We chose to exclude these patients from the comparisons. In two cases, TEE could not properly identify the puncture needle or the fossa ovalis, increasing the procedure time. Other differences between the methods include the safety and the autonomy provided by the ICE, considering it allows the same operator to perform imaging and ablation simultaneously and the need for esophageal intubation in the TEE group. The total fluoroscopy time was similar between the groups, as the factors that prolonged the procedure time using TEE cannot be compensated with greater use of X-rays. In the meta-analysis by Isath et al.,^10^which gathered 19 studies and compared the results of ablations performed with or without EIC, there was a reduction in both fluoroscopy and procedure time. However, it must be considered that a direct comparison was not made with TEE but with isolated fluoroscopy and/or electro-anatomical mapping.

TEE is the gold standard for identifying a thrombus in the LA appendix.^4^TEE is an invasive method with a complication risk of approximately 0.9%.^11 , 12^ICE can improve the procedure’s outcome by reducing the risk of recurrence of AF and the risk of pulmonary vein stenosis,^13^despite its limitation in properly identifying small branches of the pulmonary veins. Isath et al.,^10^in a 14-year analysis involving several North American centers, demonstrated that the use of ICE during AFA was related to lower in-hospital mortality, lower risk of procedure complications, and shorter hospital stay. Specifically, ICE allows for faster detection of complications, including pericardial effusion, air embolism, ventricular dysfunction as a cause of hypotension, and thrombus formation in sheaths and catheters used for ablation.

A meta-analysis by Goya et al.,^14^evaluated the results using ICE for arrhythmia ablation. Of the 19 included studies, 14 were performed in patients with AF. The use of ICE was associated with a significantly lower fluoroscopy time, fluoroscopy dose, and shorter procedure time without compromising the clinical efficacy or safety of the procedure. Although the findings are like our study, it should be noted that the meta-analysis by Goya et al.^14^was not a direct comparison between EIC and TEE. The broad comparator group included electro-anatomical mapping, fluoroscopy, or other imaging modalities, sometimes used in conjunction with or nothing in addition to fluoroscopy. Ribeiro et al.,^15^comparing percutaneous occlusion of the left atrium guided by ICE with TEE, found similar procedure time, risk of complications, and procedure time between the two methods.

There is a linear relationship between radiation dose and the risk of future malignancy. Radiation exposure is also linked to acute skin injuries, thyroid disorders, and cataracts, among other illnesses.^16 , 17^Since no dose of radiation is considered safe, and operators may be at greater risk due to the multiple procedures they perform, it is recommended that radiation exposure be as low as possible.^17^In this sense, the significantly lower dose to which patients in the ICE group were submitted is a point in favor of the method. We believe that the ICE allowed the use of images with lower intensity, which reduced the radiation dose, despite the similar fluoroscopy times.

By requiring an additional 11 Fr sheath vascular access, ICE may increase the risk of vascular complications. This was not the case in our study. In the meta-analysis performed by Goya et al.,^14^there was a trend towards an increase in vascular complications with ICE (RR 1.93; 95% CI, 0.81–4.60; p = 0.14).

ICE requires proper operator training in catheter manipulation and image interpretation. Because it is being progressively adopted in other forms of ablation,^18 , 19^we believe that it should be part of the skill set of electrophysiologists who perform complex ablations. ICE also requires a non-reusable catheter, increasing the cost of the procedure, which is at least partially offset by the reduction in the number of staff needed for the procedure and the reduction in the consumption of anesthetic medication.^20^

ICE allows real-time visualization of cardiac structures and intraprocedural assessment of the catheter-tissue interface. Our sample was insufficient to compare the procedure’s effectiveness between the two methods. In a real-world study, ventricular tachycardia ablation using ICE was associated with a lower likelihood of ventricular tachycardia-related readmission at 12 months and repeat ablation when compared to ablation without the aid of ICE.^18^A retrospective study of Medicare patients undergoing AFA found that the use of ICE was associated with a lower risk of repeat ablation (hazard ratio 0.59; 95% CI 0.37-0.92).^21^

### Study limitations

Our study suffers from some limitations. (1) Small sample size may be responsible for not detecting some differences between groups, such as length of hospitalization. (2) Lack of randomization. The use of the ICE was based on the availability of health insurance, which could systematically influence the profile of patients in each group. Table 1 shows that both groups had a similar profile regarding the main risk factors for AF recurrence and technical difficulty during ablation, including left atrial size, which mitigates this potential bias. Some explanations for the difference in procedure time between groups, such as operator availability in the TEE group, are due to local factors and may not reflect the reality of other institutions. (3) We did not perform a direct cost comparison between the two strategies. As the patients had different health plans, the form of reimbursement and the cost varied accordingly, preventing a homogeneous analysis of the effect of the method employed on the costs. The intracardiac echocardiogram is sold to our institution for R$ 7500.00. Regarding the TEE, the procedure time, on average 55 minutes longer, would increase room costs by R$ 1100.00, while the professional to perform the exam would have an average cost of R$ 1000.00. It is not possible to estimate the indirect cost of this greater occupation of health resources, especially during the pandemic, when 25 of the 49 ablations analyzed in the study were performed.

## Conclusion

In our cohort, the use of AFA with ICE was related to shorter procedure times and less exposure to radiation without increasing the risk of complications or length of hospital stay. The increase in the initial cost of the procedure can be compensated by a lower occupancy of the operating room and the need for fewer employees trained in the procedure.
